# Single-Voxel Proton Magnetic Resonance Spectroscopy of the Thalamus in Idiopathic Epileptic Dogs and in Healthy Control Dogs

**DOI:** 10.3389/fvets.2022.885044

**Published:** 2022-07-07

**Authors:** Nico Mauri, Henning Richter, Frank Steffen, Niklaus Zölch, Katrin M. Beckmann

**Affiliations:** ^1^Clinic for Diagnostic Imaging, Department of Diagnostics and Clinical Services, Vetsuisse Faculty, University of Zurich, Zurich, Switzerland; ^2^Vetimage Diagnostik GmbH, Oberentfelden, Switzerland; ^3^Section of Neurology and Neurosurgery, Small Animal Clinic, Vetsuisse Faculty, University of Zurich, Zurich, Switzerland; ^4^Department of Forensic Medicine and Imaging, Institute of Forensic Medicine, University of Zurich, Zurich, Switzerland

**Keywords:** MRS—^1^H nuclear magnetic resonance spectra, N-acetyl aspartate (NAA), glutamine (Gln), glutamate (Glu), glutamate-glutamine (Glx), canine, generalized seizures

## Abstract

The role of magnetic resonance spectroscopy (MRS) in the investigation of brain metabolites in epileptic syndromes in dogs has not been explored systematically to date. The aim of this study was to investigate metabolites in the thalamus in dogs affected by idiopathic epilepsy (IE) with and without antiepileptic drug treatment (AEDT) and to compare them to unaffected controls. Our hypothesis is that similar to humans with generalized epilepsy and loss of consciousness, N-acetyl aspartate (NAA) would be reduced, and glutamate–glutamine (Glx) would be increased in treated and untreated IE in comparison with the control group. In this prospective case–control study, Border Collie (BC) and Greater Swiss Mountain dog (GSMD) were divided into three groups: (1) healthy controls, IE with generalized tonic–clonic seizures with (2) and without (3) AEDT. A total of 41 BC and GSMD were included using 3 Tesla single-voxel proton MRS of the thalamus (PRESS localization, shortest TE, TR = 2000 ms, NSA = 240). After exclusion of 11 dogs, 30 dogs (18 IE and 12 healthy controls) remained available for analysis. Metabolite concentrations were estimated with LCModel using creatine as reference and compared using Kruskal–Wallis and Wilcoxon rank-sum tests. The Kruskal–Wallis test revealed significant differences in the NAA-to-creatine (*p* = 0.04) and Glx-to-creatine (*p* = 0.03) ratios between the three groups. The Wilcoxon rank-sum test further showed significant reduction in the NAA/creatine ratio in idiopathic epileptic dogs under AEDT compared to epileptic dogs without AEDT (*p* = 0.03) and compared to healthy controls (*p* = 0.03). In opposite to humans, Glx/creatine ratio was significantly reduced in dogs with IE under AEDT compared to epileptic dogs without AEDT (*p* = 0.03) and controls (*p* = 0.02). IE without AEDT and healthy controls did not show significant difference, neither in NAA/creatine (*p* = 0.60), nor in Glx-to-creatine (*p* = 0.55) ratio. In conclusion, MRS showed changes in dogs with IE and generalized seizures under AEDT, but not in those without AEDT. Based upon these results, MRS can be considered a useful advanced imaging technique for the evaluation of dogs with IE in the clinical and research settings.

## Introduction

Advanced neuroimaging has been recently introduced in the diagnostic work-up and research of canine epilepsy (1, 2). One of these advanced neuroimaging techniques is magnetic resonance spectroscopy (MRS). In human epilepsy, MRS is, among other techniques, widely used to rule out metabolic diseases in structural negative epilepsy (3), for presurgical evaluation (3), and to study the biochemical composition during drug treatments (4, 5). MRS studies investigating canine epilepsy are sparse. Few case reports and studies showed the utility of MRS in genetic (6) and in structural epilepsy (7, 8). However, only one study examined MRS in idiopathic epileptic dogs (9).

MRS allows non-invasive estimation of metabolite concentrations within a selected volume of interest (VOI) by exploiting slight changes in the magnetic field sensed by nuclei, usually ^1^H protons, in different metabolites. This so-called chemical shift results in specific peaks for different metabolites in a frequency spectrum (10). Metabolites that can be identified with MRS at 3 Tesla (3T) and are of specific interest in epilepsy include excitatory and inhibitory neurotransmitters, such as glutamate and gamma-amino-butyrate (GABA), but also markers for neuronal integrity, such as N-acetyl aspartate (NAA) (11).

Glutamate is the main excitatory neurotransmitter in the brain. Due to the overlap of glutamate and glutamine spectra and the difficulties in separating these two spectra at field strength lower than 3T, glutamate is commonly reported together with glutamine as glutamate–glutamine complex (Glx) (12). Excessive glutamate release is observed in chronic epilepsy and associated with recurrent seizures (13). In human epilepsy, significant changes in Glx have been identified in different brain regions depending on the underlying epileptic syndrome (11). While for temporal lobe epilepsy with hippocampal necrosis, reduced Glx was found in the ipsilateral hippocampus (14), and increased levels of Glx were detected in the medial prefrontal cortex in juvenile myoclonus epilepsy (15).

GABA is the major inhibitory neurotransmitter of the central nervous system. Furthermore, many antiepileptic drugs target the GABA system and cause increased GABA concentration in the brain (4). However, MRS evaluation of GABA is challenging. GABA concentration compared to other brain metabolites is much lower, and the peaks of GABA in MRS overlap with those of other metabolites (16). Because advanced techniques, such as spectral editing seem unavoidable to obtain reliable results at 3T, information regarding GABA levels in epilepsy is still scarce (11, 17).

NAA is one of the molecules with the highest concentration in the brain (18). Its function in the brain is still controversial and under investigation (18). NAA is mostly reported together with the neurotransmitter N-acetylaspartylglutamate (NAAG) as total NAA (tNAA) because these signals cannot be reliably separated under common conditions (19). NAA is an intraneuronal metabolite synthetized in the mitochondria. Although NAA is not only present in neurons but also in oligodendrocytes/myelin, NAA in MRS is considered the key neuronal marker for brain neuronal health, viability, and number of neurons (18). In human epilepsy, focal reductions in NAA were found in different forms of temporal lobe epilepsy (20). Furthermore, recovery of the NAA levels after successful epilepsy surgery was reported (21).

The presented clinical applications in human medicine demonstrate that alterations in brain metabolites can be detected with MRS. However, one should notice that the brain regions where these alterations can be detected, as well as the magnitude of the alterations strongly depends on the specific underlying epileptic syndrome are not uniform.

In single-voxel MRS, a VOI has to be selected. This represents a major challenge in veterinary medicine. First, because epileptic syndromes are not as well characterized in dogs as in humans (22). Second, as the main target area within the brain in canine epilepsy remains undefined and may be variable in subpopulations of dogs diagnosed with idiopathic epilepsy (IE).

To overcome the issue of the heterogenicity in canine epileptic syndromes, we decided to select breeds with rather well-characterized epileptic syndrome and familiar history of epilepsy, suggesting a common genetic background for the diseases in this breed (23). From those dog breeds presented to our hospital more commonly with IE, Border Collie (BC) and Greater Swiss Mountain Dog (GSMD) met these criteria (24, 25).

Target volume definition is more difficult to solve. One could argue that multivoxel MRS assessing the whole brain could be an alternative. However, standard multivoxel MRS at 3T suffers from long acquisition times, low spatial resolutions, and variable quality (26, 27). With regard to these problems, we decided to use single-voxel MRS, thus, facing the challenge of selecting a specific target VOI. In a recent study, Olszewska at al. selected the temporal lobe as target area in canine IE (9). However, the involvement of the temporal lobe in canine epilepsy is still controversial (9). In humans, a recent MRS study demonstrated alteration of brain metabolites in the thalamus in people affected by the loss of consciousness during seizures (28). Also, BC and GSMD are affected by generalized tonic–clonic seizures which are associated with the loss of consciousness (22). The thalamus is one of the most important center associated with the loss of consciousness in epilepsy in people (29). This together with the thalamic MRS changes detected in humans with the loss of consciousness during epileptic seizures makes the thalamus an appropriate target area for MRS evaluation also in dogs with seizures accompanied by the loss of consciousness.

The purpose of our study was therefore to assess and compare thalamic MRS spectra in healthy control dogs and in IE dogs affected by generalized seizures with a focus on NAA and Glx. Another aim of this study was to assess possible differences between IE dogs with and without antiepileptic drug treatment (IE with AEDT and IE without AEDT, respectively). Our hypothesis was that IE-affected dogs, similar to humans with seizures and loss of consciousness, show elevated Glx and reduced NAA concentration in the thalamus compared to control dogs and that significant differences in NAA and Glx could be detected between treated and untreated dogs with IE.

## Materials and Methods

### Study Population

This prospective study was performed between 2017 and 2021 at the Veterinary hospital of the University of Zurich after approval by the cantonal authorities according to Swiss law under animal license no. ZH272/16 and ZH046/20.

We recruited GSMD and BC dogs with presumptive diagnosis of IE (cases) and clinically healthy relatives, GSMD and BC (controls) of the prospectively enrolled cases. Dogs affected by IE had to fulfill the TIER II criteria for IE of the veterinary epilepsy task force (suspected genetic epilepsy) (30) and had to show generalized tonic–clonic seizures.

All dogs underwent complete clinical and neurological examination performed by a board-certified veterinary neurologist, blood screening for metabolic epilepsy, magnetic resonance imaging (MRI) of the brain, and MRS of the thalamus. Dogs with IE had additionally cerebrospinal fluid (CSF) analyses performed.

The IE dogs were divided into two groups: one group of IE dog was drug-naïve (IE without AEDT), and the other one already received AEDT (IE with AEDT).

Exclusion criteria were MRS of nondiagnostic quality, voxel placement outside of the thalamus, abnormal clinical or neurological examination, abnormal CSF analyses, or identification of an underlying cause for the epilepsy.

### Procedures

All dogs underwent MRI and MRS under general anesthesia with a standardized anesthesia protocol previously described (31). A part of the dogs included in this study has also been included in the aforementioned study.

MRI and MRS were performed with a 3T MRI (Philips Ingenia scanner, Philips AG, Zurich, Switzerland) with a 15-channel receive–transmit head coil (Stream Head-Spine coil solution, Philips AG, Zurich, Switzerland) in all patients.

Conventional morphological MR images included T2-weighted (W) turbo spin-echo sequences in transverse, dorsal, and sagittal planes, a fluid-attenuated inversion recovery (FLAIR), a T2^*^ or a susceptibility-weighted sequence, and diffusion-weighted images in transverse, as well as a 3D T1-W sequence before and after intravenous injection of contrast media [gadodiamide (Omniscan) 0.3 mmol/kg, GE Healthcare AG, Glattbrugg, Switzerland, or gadoteric acid (Dotarem) 0.3 mmol/kg, Guerbet AG, Zürich, Switzerland].

Single-voxel MRS of the thalamus was performed with a previously described optimized protocol (32). In brief, transverse, dorsal, and sagittal T2-W images were used to graphically place the single voxel in the thalamus, preferably in the right thalamus. Voxel size was 1.8 cm^3^ (10 x 12 x 15 mm), and care was taken to avoid CSF, as well as peripheral soft and bony tissues adjacent to the thalamus to prevent lipid contamination. Before MRS acquisition with point-resolved spectroscopy (PRESS) localization and water suppression using the excitation technique, field homogeneity was optimized with a second-order automatic pencil-beam shim. In addition, a water-unsuppressed spectrum was obtained as concentration reference to estimate metabolite concentrations. Spectra were obtained using the following parameters: shortest possible echo time (TE): 29 to 31 ms; repetition time (TR): 2,000 ms; number of signal averages (NSA): 240; bandwidth: 2,000 Hz. Spectra outside the thalamus and spectra with the presence of artifacts (e.g., presence of strong lipid contamination) were excluded based on visual inspection.

MRI image evaluation and visual MRS analyses were performed on each dog by a board-certified veterinary radiologist or a resident in diagnostic imaging under direct supervision of a board-certified veterinary radiologist.

### Data Processing

Metabolite concentrations were estimated, as described before (32) with an automated data processing spectral fitting algorithm (linear combination model, LCModel, version 6.3, S Provencher, Oakville, ON, Canada) using a simulated basis set (details can be found in Supplementary Table S1).

To translate the fitted MRS signals into estimates of the metabolite concentrations, an external or internal reference is needed (33). The most common internal reference used in human medicine is tissue water signal (33). Tissue water signal has the advantage of being about 10,000 times higher than the signal of the metabolites. In addition, water signal does not have to be resolved from overlapping metabolites. Nevertheless, to use reliably tissue water signal, segmentation of voxel into gray, white matter, and CSF is recommended, especially to correct for the CSF contamination in the measured voxel (33). Because tissue segmentation was not available in our study, we initially opted for total creatine (tCr, sum of creatine and phosphocreatine) as an internal reference. However, as creatine is a marker for energy metabolism, creatine ratios have the disadvantage that this metabolite can also be affected by some diseases and by the process of aging (33). Successively, we also used the water signal as reference in order to check the results obtained with the creatine ratios.

Metabolite-to-water ratios expressed in institutional units were derived within LCModel using the unsuppressed water signal with a global correction for the relaxation attenuation of the water signal (ATTH20 = 0.7) and estimation of the water concentration in the measured voxel of WCONC = 43300. Metabolite signals were not corrected for relaxation attenuation.

To assess the quality of the spectra, signal-to-noise ratio (SNR), linewidth in form of full width at half maximum (FWHM), and relative Cramér Rao lower bounds (%CRLBs) as estimation of the lower bounds of fitting error (34) were collected from the LCModel output. In addition, the FWHM of the unsuppressed water scan was measured.

Comparison of metabolite ratios and MRS spectra in the thalamus was performed between dogs affected by IE with and without AEDT and healthy control dogs.

### Statistical Analysis

Statistical analysis was performed using R (version 4.1.2 in RStudio) (35). All groups (IE without AEDT, IE with AEDT, and healthy control dogs) were compared using a non-parametric Kruskal–Wallis test on all metabolite ratios to total creatine and on all metabolite ratios to water, respectively. Pairwise comparison between groups was performed using the Wilcoxon rank-sum test. Overall, *p* < 0.05 was considered to be statistically significant.

## Results

### Study Population

Forty-one dogs fulfilled the inclusion criteria. Nine of these 41 dogs were excluded due to mispositioning of the VOI, one dog was excluded due to incorrectly set echo time during the MRS examination, and one dog was excluded due to visual identification of a broad and abnormal peak in the lipid region. Of the remaining 30 dogs, breeds were represented as follows: 20 GSMD (66.7%) and 10 BC (33.3 %). Population characteristics and seizure semiology are listed in Tables 1, 2, respectively. In the majority of the cases, the VOI for the MRS was placed in the right part of the thalamus (25; 83%) and in the remaining dogs in the left part (5; 17%).

**Table 1:** Population characteristics.

	**Healthy controls**	**IE without AEDT**	**IE with AEDT**
**Breed**			
BC	2	2	6
GSMD	10	7	3
**Sex**			
Male	8	6	4
Male castrated	-	-	1
Female	4	3	-
Female spayed	-	-	4
**Bodyweight**			
kg (median, range)	47.5, 20–55	48, 14–70	21, 15–60
**Age**			
Years (median, range)	5.4, 1.3–8.1	4.7, 1–7, 4	5.3, 3.3–7

*IE, idiopathic epilepsy; AEDT, antiepileptic drug treatment; BC, Border Collie; GSMD, Great Swiss Mountain Dog*.

**Table 2:** Semiology of epileptic events in the affected population.

	**IE without AEDT**	**IE with AEDT**
**Seizures**		
Status epilepticus	-	1
Cluster seizures	-	5
**Seizure semiology**		
Tonic-clonic	9	9
Tonic	-	-
Focal onset secondary generalization	3	3
Unknown onset	6	6
Additional focal seizures	-	-
Autonomic signs	4	5
**Time between first seizure to MRI**		
<1 month	-	-
>1–3 month	3	1
>3–12 month	4	3
>12 months	-	2
>24 months	2	3
**Time between last reported seizure to MRI**		
2–7 days	2	2
8–31 days	5	5
>1 month	2	2
**Medical treatment at the timepoint of MRI**		
Phenobarbital	-	9
Potassium bromide	-	2
Levetiracetam	-	3
Imepitoin	-	3
Gabapentin	-	1
**Special diet or dietary supplement before MRI**		
Medium chain triglycerides	1	1
Cannabidiol	1	1

*IE, idiopathic epilepsy; AEDT, antiepileptic drug treatment*.

### Spectral Quality

**Figure 1:**
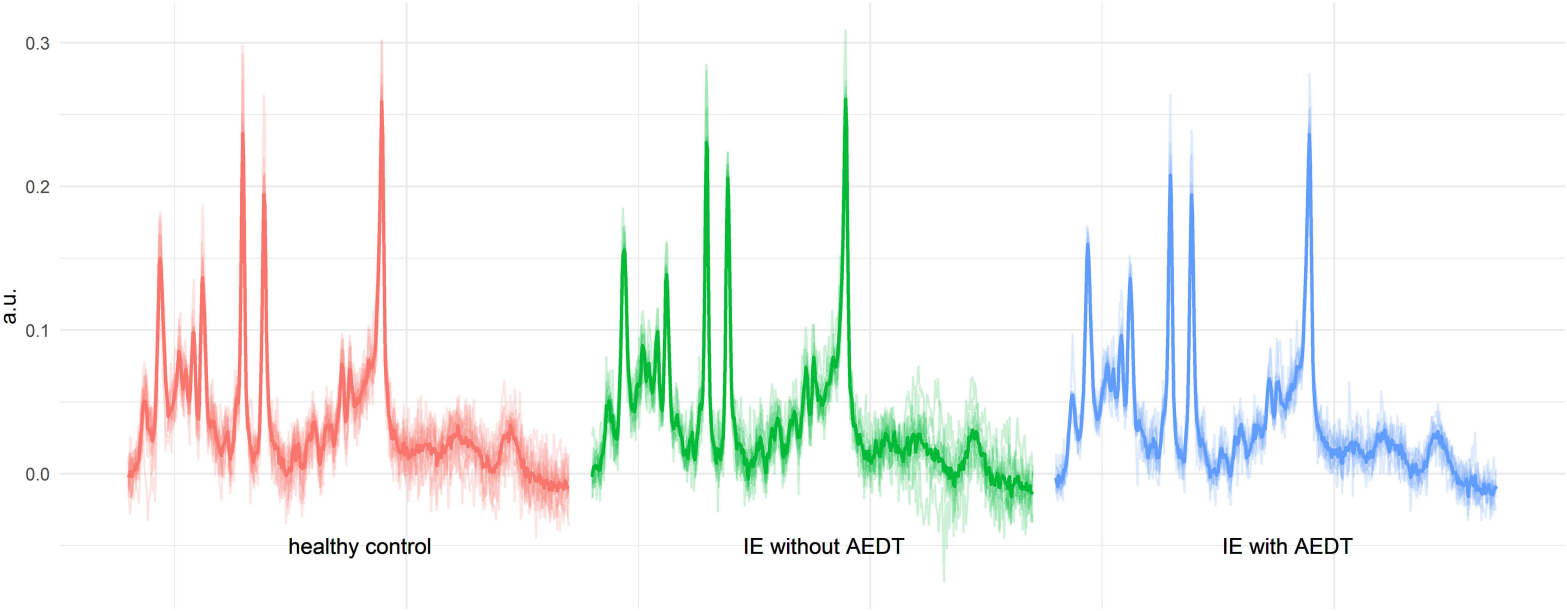
All measured spectra of the three investigated groups. In the background, the individual spectra of all dogs are plotted as outputted from LCModel (.coord files). The thicker line shows the calculated mean spectrum in each group. The spectra show a consistent quality, and the observed differences in N-acetyl aspartate (NAA) and glutamate–glutamine (Glx) in idiopathic epilepsy (IE) with antiepileptic drug treatment (AEDT) are already indicated in this visual overview.

**Figure 2:**
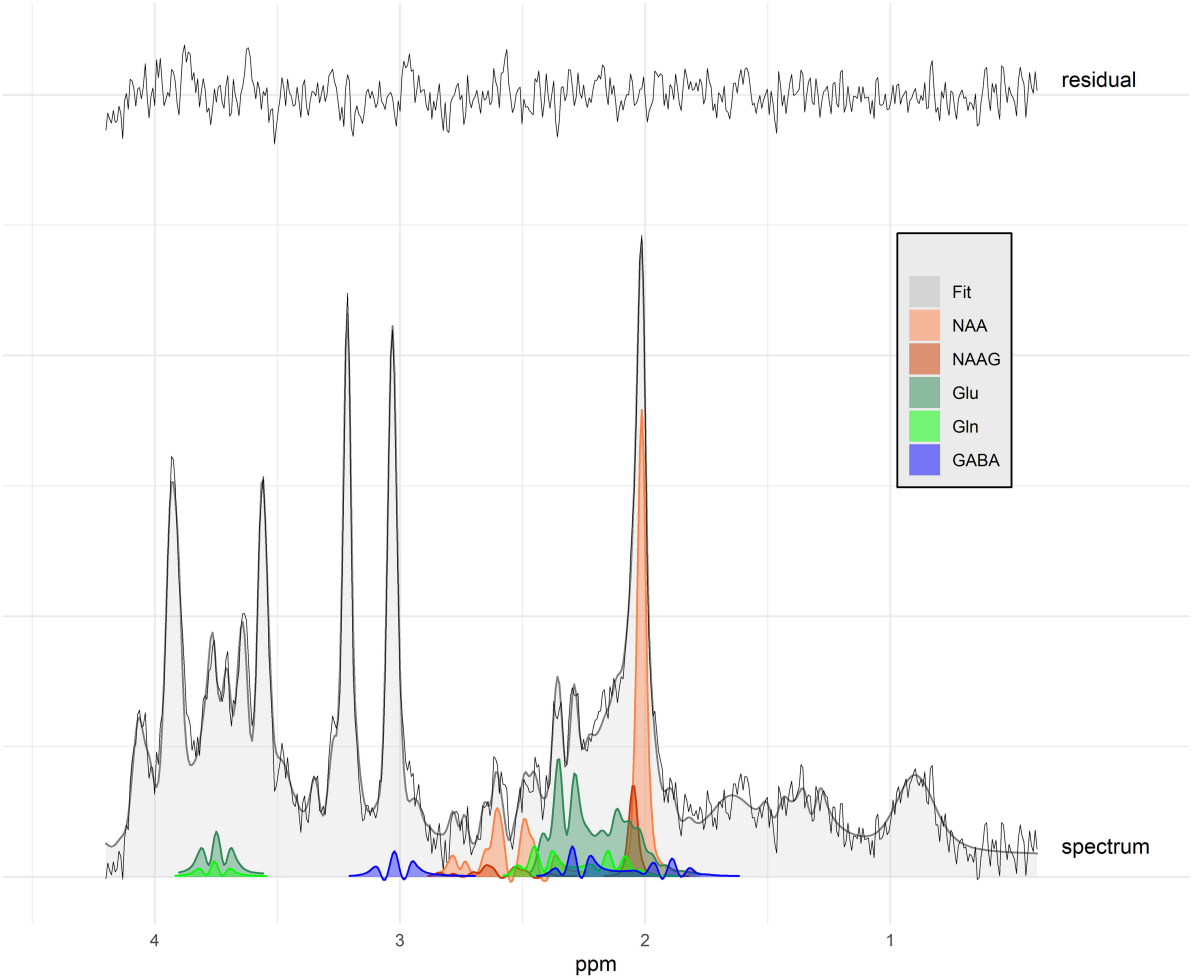
Spectrum with signal-to-noise ratio of 16 (study mean: 12.9) and water peak full width at half maximum of 6.8 Hz (study mean: 6.79 Hz) of an idiopathic epileptic dog with antiepileptic drug treatment. The black line shows the acquired data, the gray area the overall fit, and the colors the individual fit components displayed for selected metabolites. Fit residuals are shown at the top. NAA, N-acetyl aspartate; NAAG, N-acetylaspartylglutamate; Glu, glutamate; Gln, glutamine; GABA, gamma-amino-butyrate.

All spectra obtained were of good quality, with no spectra being rejected (Figure 1). The SNR in the study was between 19 and 8, and the FWHM of the fit (LCModel Output) was 2.9–5.9 Hz (Figure 2). No significant differences between the groups were found for these quality parameters. However, the FWHM of the water peak (between 5.6 and 7.8 Hz) was slightly higher (0.5 Hz) in the treated group.

### MRS Results

The metabolite-to-total creatine ratios of all measured metabolites and the %CRLB are listed in the Supplementary Table S2.

**Figure 3:**
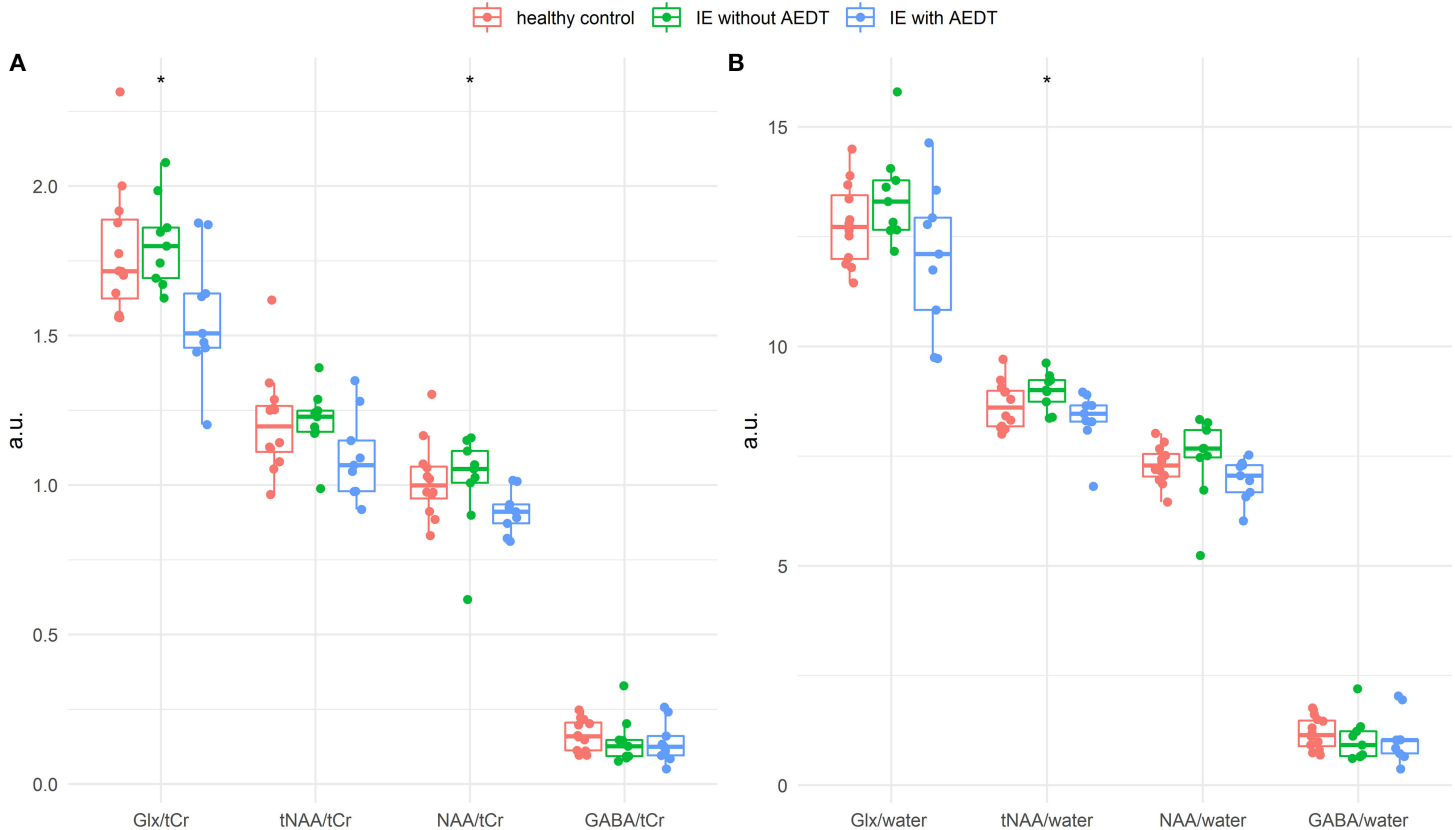
Box plot of thalamic metabolite concentrations relative to creatine **(A)** and water **(B)** in healthy control dogs (red), dogs affected by idiopathic epilepsy with antiepileptic drug treatment (IE with AEDT in blue), and non-treated epileptic dogs (IE without AEDT in green). The Kruskal–Wallis test revealed statistically significant difference in the metabolites NAA/creatine and Glx/creatine, as well as tNAA/water (asterisk). Glx, glutamate–glutamine complex; tCr, total creatine; tNAA, total NAA; NAA, N-acetyl aspartate; GABA, gamma-amino-butyrate.

The Kruskal–Wallis test revealed significant differences in the NAA-to-creatine (*p* = 0.04) and Glx-to-creatine (*p* = 0.03) ratios between the three groups. Significant differences were also found in the tNAA-to-water ratios (*p* = 0.04) (Figure 3).

Pairwise comparisons showed significant decrease in NAA-to-creatine ratios in IE dogs under AEDT compared to IE dogs without AEDT (*p* = 0.03) and compared to healthy control dogs (*p* = 0.03). However, tNAA-to-creatine ratio was not significantly reduced between the IE dogs with AEDT compared to IE without AEDT (*p* = 0.06) nor compared to healthy control dogs (*p* = 0.17).

Glx-to-creatine ratio was significantly reduced in IE dogs under AEDT compared to IE dogs without AEDT (*p* = 0.03) and compared to healthy dogs (*p* = 0.02).

Pairwise comparison of IE without AEDT and healthy controls did not show significant difference, neither in NAA-to-creatine (*p* = 0.60), nor for tNAA-to-creatine (*p* = 0.81) or for Glx-to-creatine (*p* = 0.55) ratio.

NAA and tNAA-to-water ratios were significantly reduced in IE dogs with AEDT compared to IE dogs without AEDT (*p* = 0.04; *p* = 0.01, respectively) but not compared to healthy dogs. Glx to water was not significantly reduced between the compared latter groups of IE dogs (*p* = 0.08).

## Discussion

In human epilepsy, MRS has been introduced into the work-up since more than two decades. In contrast, MRS application in canine epilepsy is still in its early days. We investigated thalamic metabolite concentrations with MRS in healthy control dogs and IE dogs with and without AEDT. It was hypothesized that dogs affected by IE showed elevated Glx and reduced NAA concentration in the thalamus compared to control dogs, similar to humans with seizures and loss of consciousness (28). In this study, dogs with IE under AEDT, but not those without AEDT, had significantly lower NAA-to-creatine ratios compared to healthy controls, as well as compared to IE dogs without AEDT. With also reduced tNAA in IE with AEDT, this would convey a relatively clear picture. However, although the tNAA-to-creatine ratio is also generally lower in dogs with IE on AEDT, the differences are not significant. NAA and NAAG are spectroscopically very similar (19), as can also be seen in Figure 2. Thus, a distinction at 3T is difficult and strongly dependent on the spectral quality. With linewidths of <8 Hz obtained herein, which is considered excellent for measurements in humans (36), a reliable assignment seems at least plausible. In this case, the absence of differences in tNAA could be explained by changes in NAAG. However, given the observed differences in water linewidth, we must at least consider that the observed difference in NAA could reflect the difference in linewidth and thus a biased fitting. The measured ratios to water confirm the differences for NAA between IE dogs under AEDT compared to IE dogs without AEDT, but there are no significant differences in NAA or tNAA water ratios to the healthy dogs, which do not simplify the interpretation of the results.

Reduction in NAA in MRS is considered a sign for neuronal loss (18), and several factors can contribute to decreased NAA levels in human epilepsy. Some of these factors may also explain the lower NAA-to-creatine ratio found in IE dogs under AEDT compared to those without AEDT.

The number of seizures is an important reason for reduced NAA levels. In humans with generalized tonic–clonic seizures, reduction in NAA was more severe in patients who experienced more than ten generalized tonic–clonic seizures during their lifetime, than in patients with less seizure episodes (37–39). In our study, the total number of seizures per dog was not available, but overall longer duration of epilepsy and more common occurrence of cluster seizures and status epilepticus in dogs with AEDT compared to those without AEDT, suggests a higher number of seizures in this group. Therefore, the NAA reduction in IE dogs with AEDT might reflect the findings in humans, where patients with more seizure episodes showed severer NAA diminution (37–39).

Another reason for reduced NAA levels is poor seizure control. In humans, lower NAA levels were found in the mesial temporal lobe in patients failing to respond to the first AEDT compared to those with seizure freedom after first AEDT (40). Of the nine dogs in our study with AEDT, six had more than one antiepileptic drug. The high number of cases with multidrug treatment suggests a high level of resistance to the first antiepileptic drug in this group. One possible explanation in humans for lower NAA in non-responders is more severe neuronal damage in this group compared to responders (41). We may speculate that this is also the case in IE dogs with multidrug AEDT in our study. Longitudinal MRS studies during the course of epilepsy with comparison of MRS results between first drug responders and non-responders could help to answer this question.

The third factor resulting in decreased NAA levels is the disease duration and aging. Decreased NAA in humans has been reported with aging in medical resident temporal lobe epilepsy and with increased duration of epilepsy in secondary generalized tonic–clonic seizures bilaterally in the thalamus (28, 42). In our population, dogs under AEDT had slightly higher median age and longer disease duration compared to IE dogs without AEDT. This may also have contributed to the NAA changes, similarly as in human medicine.

Little is known about NAA ratios in dogs suffering from IE. The only study investigating MRS in canine IE was performed with magnetic field strength of 1.5T, MRS VOI placement in the temporal lobe, and included only drug-naïve dogs, without reporting seizure frequency or severity (9). Similar to our findings in IE dogs without AEDT, in the study of Olszewska et al., no significant differences were identified in either NAA/Cr, NAA/choline (Cho), or Cho/Cr between drug-naïve IE dogs and control dogs (9). Interestingly, Olszewska and colleagues found a temporal correlation between NAA/Cho and Cho/NAA ratios and time elapsed between MRS scan and the last reported seizure episode. Higher NAA/Cho ratios were present in dogs with very recent seizure activity, in contrast to lower ratios in dogs with less recent seizure episodes (9). In our study, we included only dogs with a gap of at least 2 days between last reported seizure event and MRI/MRS scan. In addition, interval between last reported seizure and MRI/MRS was similar in IE dogs with and without AEDT. Thus, it seems unlikely that the lower NAA ratios detected in our study were influenced by the time interval between the last seizure episode and the time of scanning. It is beyond the scope of this study to draw definitive conclusions about the cause of reduced NAA. Pathological studies systematically proving evidence for neuronal loss in canine epilepsy are lacking (43, 44), but early onset of mental decline/cognitive dysfunction supports impaired neuronal integrity in dogs suffering from IE (45).

In contrast to our hypothesis, we did not find an increase, but rather a decrease in thalamic Glx-to-creatine ratios in dogs with IE under AEDT compared to healthy controls and cases without AEDT.

Even relatively small differences in linewidth have been shown to bias estimates of Glx (46). The linewidth of the water peak was 0.5 Hz higher in IE dogs under AEDT, than in the other groups, and we cannot exclude an influence of the different linewidths on the Glx results. The Glx ratios to water show a similar picture as in the case for NAA. While the difference between IE dogs with and without AEDT stays visible, the difference in the healthy control disappears. This indicates possible differences in the reference signals total creatine or water between the healthy and the IE dogs, which weaken or amplify the differences.

Glx is the sum of glutamine and glutamate, which are present in both neuronal and glial cells. Concentrations of glutamate and glutamine are coupled *via* glutamate–glutamine cycling. Slow rates of glutamate–glutamine cycling, reduced glutamine levels, and a relative increase in glutamate levels have been found in resected epileptogenic hippocampi (47). Glx-to-creatine ratios in human epilepsy depend on the epileptic syndrome investigated and the area of interest within the brain and are not without controversy. While for example increased Glx-to-creatine ratios have been reported in the thalamus in juvenile myoclonic epilepsy, decreased levels have been reported in the frontal cortex (48). Another study investigating the prefrontal cortex in juvenile myoclonus epilepsy found also increased Glx-to-creatine ratios in the thalamus but decreased ratios in the medial prefrontal cortex, an area anatomically very close to the frontal cortex (15). A decrease in Glx is also considered an early biomarker of neuronal degeneration (49), and reduced frontal cortex Glx ratios have been linked to deficient frontal lobe functions in juvenile myoclonus epilepsy (48). No consistent effects of different types of AEDT on Glx levels in humans have been reported (4). It is assumed that antiepileptic drugs do not alter the glutamate concentration directly, but instead decrease the sensitivity of the glutamate receptor. A negative modulation of the voltage-gated channels might then lead to decreased glutamate concentrations (4). So far, no studies have been conducted on the effect of antiepileptic drugs on Glx concentrations in dogs, making it impossible to distinguish the effect of the drug from the effect of the disease on the Glx results in or study. Remarkably, potential contribution of AEDT associated lower glutamate levels to suboptimal cognitive functioning in patients with epilepsy has been stated (5). Cognitive decline has also been reported in canine IE, with dogs showing cluster seizures and higher seizure frequency having more severe cognitive decline (45). In our study, IE dogs with AEDT had more often history of cluster seizures than those without AEDT. Therefore, having a higher risk for cognitive declines. However, because cognitive function has not been systematically investigated in our study, we cannot draw definitive conclusion on this aspect, and it remains unclear whether lower Glx levels also reflect lower cognitive function in our study population. Glx is difficult to detect with long echo time MRS at 1.5 T, and therefore, the only study investigating canine IE so far has not reported Glx levels (9). Glutamate levels have been measured in the CSF of IE dogs, and CSF levels of this metabolite have been reported as increased in drug-naïve dogs with IE, as well as in dogs under AEDT (50). On the first sight, these results may look contradictory to our data. However, as highlighted before, with the MRS technique applied in this study, one is not able to measure glutamate separately from glutamine. Therefore, the decrease in Glx could rather reflect a reduction in glutamine than one glutamate. Moreover, MRS detects the intra- and extracellular Glx pool. As the intracellular concentration of glutamate greatly exceeds the extracellular glutamate concentration, small increases in the extracellular glutamate concentration can be easily overlaid by intracellular changes (51). In human medicine, impairment of the glutamate–glutamine cycle has been identified in epilepsy, but as we only measured the sum of both metabolites we cannot make conclusions on this aspect. In future, these limitations may be overcome using magnetic field strength higher than 3T or utilizing advanced MRS techniques, which allows an improved separate evaluation of glutamate and glutamine (11).

Beside NAA and Glx, the third major brain metabolite in epilepsy which can be detected by MRS is GABA. GABA is not only the major inhibitory neurotransmitter in the brain, but it is also directly connected to Glx *via* the glutamate–glutamine cycle (11). Moreover, dogs under AEDT received medications with a GABAergic mechanism of action. GABAergic antiepileptic drugs have been shown to increase the GABA concentration in MRS in humans, and GABA concentration has been positively associated with seizure control in humans (4). In our study, GABA was detected in our MRS spectra and we could not identify any significant difference between healthy controls and IE dogs with and without AEDT, respectively. However, a mean %CRLB of GABA of more than 50% clearly demonstrates the difficulty of measuring GABA with the setup used and precludes definitive conclusions. This limitation is partially due to the much lower GABA concentration in comparison with other brain metabolites and the GABA is obscured by signals from more abundant metabolites. Measurements at higher magnetic field strengths and dedicated sequences could help to overcome this technical issue (52).

**Figure 4:**
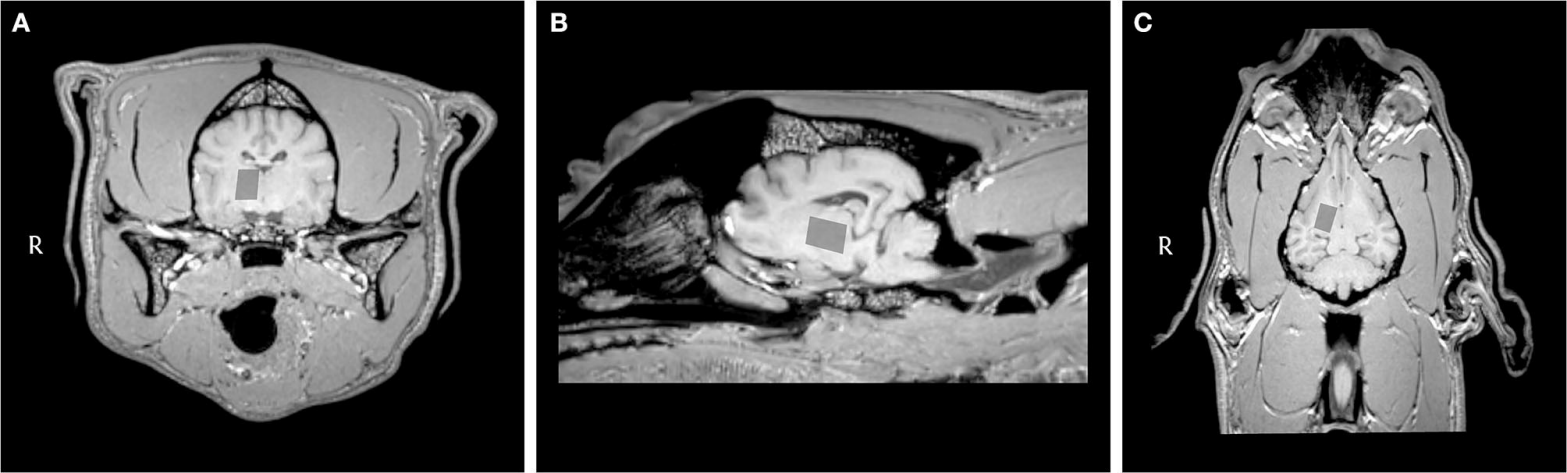
Transverse **(A)**, sagittal **(B)**, and dorsal **(C)** T1-weighted MRI images of the brain of a dog with idiopathic epilepsy showing the location of the voxel of interest in the right part of the thalamus.

In this study, we selected our VOI based on human studies in patients suffering from idiopathic generalized epilepsy, which showed significantly different metabolite concentrations primarily in the thalamus (38, 39, 53). In addition, in humans, the thalamus is one of the most important center associated with the loss of consciousness in epilepsy. This also makes the thalamus region interesting for canine IE, which is associated with the loss of consciousness (29). Furthermore, the planning of the VOI in the thalamus is straightforward and contaminations from other tissue types can be mostly avoided despite the small size of the dog brain (Figure 4). In epileptic dogs, the thalamus has not been an area of major interest so far. One perfusion study indicated differences in perfusion in the brain of IE dogs compared to healthy control dogs, among others, also in the thalamus (54). Additional support for thalamic involvement has recently been reported by Unger et al. that found bilateral rotary saturation effects in the thalamus using phase-cycled stimulus-induced rotary saturation sequence in an Old English Bulldog with generalized tonic–clonic seizures (55). Even peri-ictal MRI changes have been found in parts of the thalamus in dogs (56). Network analysis of peri-ictal lesions has shown high correlation with cingulate lesions. Combining MRS of the thalamus with MRS in the cingulate gyrus could therefore be one approach for further studies. Although the role of the thalamus in canine IE has still to be elucidated, our study is in line with previous studies and supports the involvement of this brain region in the pathogenesis of canine IE. However, the involvement of the thalamus does not allow conclusions about a possible epilepsy focus. To locate seizure-onset zones, combining MRS with electroencephalography or advanced imaging techniques, such as phase-cycled stimulus-induced rotary saturation could improve diagnostic sensitivity (2, 55).

The limitations of the study include a small sample size and inhomogeneity of the study population. In spite of finite access to cases and healthy controls, this limitation is in balance with expected number of accessible cases per year and in line with animal welfare aspects that reduce the number of experimental animals used. The study population was heterogenous in aspect of (1) IE dogs with and without AEDT, (2) different breeds of dogs, and (3) dogs not matched for age and sex. It would have been necessary to include drug-naïve dogs only, to eliminate a potential bias due to medical treatment on the MRS results. On the contrary, this would have been a preselection criterion influencing the population to newly diagnosed epileptic dogs or dogs with a milder course of the disease, which does not require AEDT. Another limitation is the fact that we evaluated the thalamus only on one side what excludes a side-by-side comparison and does not allow conclusions about possible lateralization. The choice to use the right thalamus was completely arbitrary and not supported by clinical signs. Nevertheless, we cannot exclude that measuring in the minority of the cases the left thalamus instead of the right could have influenced our results. Until now, a potential influence of the dog breed on MRS results has not been investigated. Different breeds have different skull conformations, and this also affects the shape and size of the area of interest in the brain. These anatomical differences could lead to different tissue sampling with a standardized VOI (e.g., different ratios of gray and white matter and even contamination of the VOI with CSF). Thus, we cannot fully exclude a potential bias based on this effect (33). As the study population was not matched for age and sex, we cannot rule out a potential effect on our measurements. Pairwise comparison between the treatment groups was not corrected for multiple comparison. Considering the set of statistical inferences, this seems to be balanced with the fact that comparing three groups is the lowest level of multiple comparisons possible. All these points probably contributed to the scatter of the results, and additional examinations on a larger and more homogeneous study population may help to get more precise answers.

In our study, MRS was used in research setting to assess advanced MRI techniques. However, it has been demonstrated that MRS, also in veterinary medicine, can be useful in a clinical approach (6–8, 57). Therefore, MRS should be considered as routine supplementary tool in selected patients together with conventional MRI to permit an improvement in diagnostic sensitivity. In human epilepsy research, it was suggested that future MRS studies should include MRS techniques that allow differentiation between glutamate and glutamine and to also measure GABA (11). Possibly, the availability of MRI scanners with higher field strength and the availability of new sequences will also allow this in canine epilepsy research. Furthermore, longitudinal studies with a larger and homogenous study population could improve and unfold the potential of MRS in the investigation of IE in veterinary medicine. This might help to develop biomarkers for drug response in (canine) IE and could strengthen the dog as model for the human epilepsy.

In 2015, the veterinary epilepsy task force has introduced MRI guidelines for canine IE, but these guidelines do not specify the use of MRS (1). Such consensus recommendation exists for MRS methods in humans and rodents (58, 59). By addressing MRS in canine epilepsy and applying human recommendations for reporting of methods (Supplementary Table S1) and results, we also aimed to improve standardization in MRS in veterinary medicine and we hope that in the next update of the veterinary epilepsy task force, imaging guidelines for MRS in IE will be included (60).

## Conclusion

Using MRS, we have detected reduced NAA-to-creatine ratios in IE dogs with AEDT compared to healthy controls and IE dogs without AEDT, as well as reduced Glx-to-creatine ratios in IE dogs under AEDT compared to IE dogs without AEDT. MRS can be considered as an additional imaging technique to characterize disease severity and an additional tool for canine epilepsy research, but technical limitations have to be kept in mind when interpreting the results. Further studies are needed to improve and unfold the potential of MRS in the investigation of IE in veterinary medicine and possibly create a canine model for the study of epilepsy with MRS.

## Data Availability Statement

The original contributions presented in the study are included in the article/Supplementary Material, further inquiries can be directed to the corresponding author/s.

## Ethics Statement

The animal study was reviewed and approved by the Cantonal Authorities according to Swiss Law under Animal License Nos. ZH272/16 and ZH046/20. Written informed consent was obtained from the owners for the participation of their animals in this study.

## Contribution to the Field

Magnetic resonance spectroscopy (MRS) is an advanced neuroimaging technique, which allows non-invasive estimation of metabolite concentrations within a selected volume of interest. Although MRS is extensively investigated in human epilepsy and rodent epilepsy models, MRS information in canine epilepsy is scarce. The aim of our study was to assess and compare thalamic MRS in healthy control dogs and in idiopathic epileptic dogs affected by generalized seizures, as well as to assess possible differences between idiopathic epileptic dogs with and without antiepileptic drug treatment. Significant differences, similar but not in complete accordance with MRS studies in human medicine, were detected between the investigated canine groups. In this article, we discuss these novel results and the possible reasons for the discrepancies between human and veterinary medicine. With our study, we encourage the use of MRS in canine epilepsy research and propose to add a standardized MRS protocol in the magnetic resonance imaging guidelines for canine idiopathic epilepsy, similar to the consensus recommendation approved for MRS application in humans and rodents. Further studies are needed to improve and unfold the potential of MRS in the investigation of idiopathic epilepsy in veterinary medicine, which may be used as canine model for the study of epilepsy with MRS.

## Author Contributions

KB, HR, and NZ designed the study. KB and FS collected clinical data. NM collected the MRS data and drafted the initial manuscript. NZ selected the MRS protocol and performed the LCModel and the statistical analysis. All authors edited the manuscript and approved the final manuscript.

## Funding

This research was partially financially supported by the Albert-Heim-Stiftung and the Stiftung für Kleintiere der Vetsuisse-Fakultät Universität Zürich.

## Conflict of Interest

NM was employed by Vetimage Diagnostik GmbH. The remaining authors declare that the research was conducted in the absence of any commercial or financial relationships that could be construed as a potential conflict of interest.

## Publisher's Note

All claims expressed in this article are solely those of the authors and do not necessarily represent those of their affiliated organizations, or those of the publisher, the editors and the reviewers. Any product that may be evaluated in this article, or claim that may be made by its manufacturer, is not guaranteed or endorsed by the publisher.

## Abbreviations

BC: Border Collie
Choline: Cho
CRLBs: Cramér Rao lower bounds
GABA: Gamma-aminobutyric acid
Glx: glutamate–glutamine complex
GSMD: Greater Swiss Mountain Dog
Hz: Hertz
IE: idiopathic epilepsy or idiopathic epileptic
LCModel: linear combination model
MRI: magnetic resonance imaging
MRS: magnetic resonance spectroscopy
NAA: N-acetyl aspartate
NAAG: N-acetylaspartylglutamate
NSA: number of signal averages
PRESS: point-resolved spectroscopy
SNR: signal-to-noise ratio
T: Tesla
VOI: volume of interest.
