# Supplementary material for: Single-Voxel Proton Magnetic Resonance Spectroscopy of the Thalamus in Idiopathic Epileptic Dogs and in Healthy Control Dogs
Source: Front Vet Sci. 2022 Jul 7;9:885044. doi: 10.3389/fvets.2022.885044 (PMC9302964; doi:10.3389/fvets.2022.885044)
Supplement: Supplementary file 1 [file Table_1.pdf]

Supplementary table S1: Details single voxel ^{1}H-MRS study

**1. Hardware**	
a. Field strength in Tesla [T]	3 T
b. Manufacturer	Philips
c. Model (software version if available)	Ingenia (R 5.2)
d. Radiofrequency coils: nuclei (transmit/receive), number of channels, type, body part	15 channel receive-transmit head coil (Stream Head-Spine coil solution)
e. Additional hardware	N/A
**2. Acquisition**	
a. Pulse sequence	PRESS (point resolved spectroscopy)
b. Volume of Interest (VOI) locations	Thalamus
c. Nominal VOI size [cm^3, mm^3]	10 x 12 x 15 mm^3 (APxRLxFH)
d. Repetition Time (TR), Echo Time (TE) [ms, s]	TR 2000 ms, shortest TE: 29 to 31 ms
e. Total number of Excitations or acquisitions per spectrum<br>In time series for kinetic studies<br><br>i. Number of Averaged spectra (NA) per time-point<br>ii. Averaging method (e.g. block-wise or moving average)<br>iii. Total number of spectra (acquired / in time-series)	240 acquisitions per spectrum
f. Additional sequence parameters (spectral width in Hz, number of spectral points, frequency offsets)<br>If STEAM:, Mixing Time (TM)<br>If MRSI: 2D or 3D, FOV in all directions, matrix size, acceleration factors, sampling method	Spectral width 2000 Hz, 1024 points
g. Water Suppression Method	Excitation
h. Shimming Method, reference peak, and thresholds for “acceptance of shim” chosen	Second-order automatic pencil-beam shim

i. Triggering or motion correction method (respiratory, peripheral, cardiac triggering, incl. device used and delays)	N/A
**3. Data analysis methods and outputs**	
a. Analysis software	LCModel (Version 6.3)
b. Processing steps deviating from quoted reference or product	Processing of .sdat / .spar provided by Philips with<br>a) Residual water filter ; b) 1 Hz Gaussian Filter<br>c) FWHM of water peak calculation
c. Output measure<br>(e.g. absolute concentration, institutional units, ratio)	Ratio to total creatine and to water. The ratio to water is obtained with the LCModel defaults for WCONC (water concentration in the voxel) and ATTH20 (water attenuation factor), see below. With that it is a rough estimate of moles of metabolite per volume of brain tissue (mol/L).
d. Quantification references and assumptions, fitting model assumptions	The unsuppressed water peak was used as reference with WCONC = 43300 and ATTH20 = 0.7 set within in LCModel. 20 Metabolites included in the simulated basis set (alanine, aspartate, glucose, creatine, phosphocreatine, glutamine, glutamate, glycerophosphocholine, phosphocholine,lactate, mI, NAA,N-acetyl-aspartyl-glutamate, scyllo-inositol, glutathione, taurine, glycine, phosphoethanolamine , ascorbate, and γ-aminobutyric acid.<br>Simulated contribution of macro molecules and lipid signals provided within LCModel.
**4. Data Quality**	
a. Reported variables<br>(Signal to noise ratio (SNR), Linewidth (with reference peaks))	SNR (Output from LCModel), full width at half maximum (FWHM) of the water peak
b. Data exclusion criteria	Voxel placement outside of the thalamus, visual inspection for artifacts
c. Quality measures of postprocessing Model fitting<br>(e.g. Cramér Rao lower bounds (CRLB), goodness of fit, standard deviation of residual)	%CRLBs of selected metabolites
d. Sample Spectrum	Figure 2
