# Supplementary material for: Single-Voxel Proton Magnetic Resonance Spectroscopy of the Thalamus in Idiopathic Epileptic Dogs and in Healthy Control Dogs
Source: Front Vet Sci. 2022 Jul 7;9:885044. doi: 10.3389/fvets.2022.885044 (PMC9302964; doi:10.3389/fvets.2022.885044)
Supplement: Supplementary file 2 [file Table_2.pdf]

Supplementary table S2: Metabolite concentration ratios to total Creatine

	Control (n=12)		Case (n=9)		Treated (n=9)	
	Mean	*SD*	*Mean*	*SD*	*Mean*	*SD*
**tCho**	0.36	0.05	0.35	0.06	0.33	0.06
**%CRLB**	4	0.7	4.4	0.9	3.3	0.5
**tNAA**	1.21	0.17	1.21	0.11	1.1	0.14
**%CRLB**	4.4	0.7	4.3	1	3.9	0.8
**mI+Gly**	1.18	0.11	1.11	0.11	1.17	0.1
**%CRLB**	4.8	0.9	5	1.2	3.8	1.1
**NAA**	1.02	0.13	1.01	0.17	0.91	0.07
**%CRLB**	5.2	0.8	5.6	1.4	5	1.1
**Glx**	1.78	0.22	1.81	0.15	1.57	0.21
**%CRLB**	7.6	1.1	7.3	1.1	7.1	1.6
**mI**	1.01	0.12	0.89	0.17	0.98	0.14
**%CRLB**	9.1	3.3	11.2	3.3	7.8	3.3
**Glu**	1.33	0.19	1.3	0.11	1.16	0.24
**%CRLB**	9.1	1.7	9.3	1.7	8.9	2.5
**Gln**	0.45	0.08	0.51	0.15	0.4	0.15
**%CRLB**	26.1	3.9	24	4.4	26.4	8.2

**Asp**	0.46	0.13	0.48	0.12	0.44	0.09
**%CRLB**	29.2	15.2	25.9	9	24.2	7.5
**NAAG**	0.19	0.07	0.2	0.09	0.18	0.08
**%CRLB**	30.8	14.6	29.3	12.5	28.4	15.6
**GSH**	0.18	0.1	0.19	0.05	0.13	0.05
**%CRLB**	36.6	26.8	27.9	6.1	35.3	16.7
**GABA**	0.16	0.05	0.14	0.08	0.14	0.07
**%CRLB**	51.1	17.9	60.6	20.3	60	29.3
**Asc**	0.48	0.21	0.46	0.07	0.5	0.09
**%CRLB**	102.2	282.6	22.4	6	17.3	5.4
**Scyllo**	0.03	0.02	0.02	0.02	0.02	0.02
**%CRLB**	136.8	274	251.6	378.1	182.3	314
**Glc**	0.17	0.12	0.17	0.05	0.18	0.06
**%CRLB**	155.8	290.9	41.6	23.3	33.3	12.9
**PE**	0.32	0.25	0.19	0.1	0.24	0.13
**%CRLB**	160.4	301.2	173.8	312.2	151.3	319
**Gly**	0.17	0.16	0.22	0.12	0.2	0.14
**%CRLB**	246.8	364.7	58.1	32.9	165.9	323
**Lac**	0.09	0.1	0.03	0.04	0.06	0.06
**%CRLB**	453.2	482.1	565.8	421	316.2	393.6

**Tau**	0.05	0.07	0.04	0.05	0.03	0.02
**%CRLB**	557.7	470.2	594	480.4	486.7	400.7
**Ala**	0.03	0.04	0.06	0.06	0.06	0.05
**%CRLB**	587.9	434.2	410.9	447.3	391.4	456.9

Legend: tCho: total choline; CRLB: Cramér Rao lower bounds; tNAA: total N-acetyl aspartate; mI: myoinositol; Gly: glycine; NAA: N-acetyl aspartate; Glx: glutamate-glutamine complex; Glu: glutamate; Gln: glutamine; Asp: aspartate; NAAG: N-acetylaspartylglutamate; GSH: glutathione; GABA: gamma-amino-butyrate; Asc: ascorbate; Scyllo: scyllo-inositol; Glc: glucose; PE: phosphoethanolamine; Lac: lactate; Tau: taurine; Ala: alanine.
